# SARS-CoV-2 Entry Inhibitors: Small Molecules and Peptides Targeting Virus or Host Cells

**DOI:** 10.3390/ijms21165707

**Published:** 2020-08-09

**Authors:** Rolando Cannalire, Irina Stefanelli, Carmen Cerchia, Andrea R. Beccari, Sveva Pelliccia, Vincenzo Summa

**Affiliations:** 1 Department of Pharmacy, University of Napoli “Federico II”, via D. Montesano 49, 80131 Napoli, Italy; rolando.cannalire@unina.it (R.C.); irina.stefanelli@unina.it (I.S.); carmen.cerchia@unina.it (C.C.); sveva.pelliccia@unina.it (S.P.); 2 Dompé Farmaceutici SpA, via Campo di Pile, 67100 L’Aquila, Italy; andrea.beccari@dompe.com

**Keywords:** SARS-CoV-2 entry inhibitors, COVID-19, coronavirus, spike, TMPRSS2, cathepsins, small molecules inhibitors, peptides inhibitors

## Abstract

The pandemic evolution of SARS-CoV-2 infection is forcing the scientific community to unprecedented efforts to explore all possible approaches against COVID-19. In this context, targeting virus entry is a promising antiviral strategy for controlling viral infections. The main strategies pursued to inhibit the viral entry are considering both the virus and the host factors involved in the process. Primarily, direct-acting antivirals rely on inhibition of the interaction between ACE2 and the receptor binding domain (RBD) of the Spike (S) protein or targeting the more conserved heptad repeats (HRs), involved in the membrane fusion process. The inhibition of host TMPRSS2 and cathepsins B/L may represent a complementary strategy to be investigated. In this review, we discuss the development entry inhibitors targeting the S protein, as well as the most promising host targeting strategies involving TMPRSS2 and CatB/L, which have been exploited so far against CoVs and other related viruses.

## 1. Introduction

The World Health Organization (WHO) prioritized infectious diseases as a major health issue, despite the enormous advance in the scientific knowledge of the biology and the treatment options of infections. Nowadays, viruses infections represent a major cause of human diseases with additional implications also in human cancers, neurological, and chronic disorders. The development of specific inhibitors is the key point to obtain effective antiviral drugs. One the most relevant alert is related to the RNA viruses, such as Middle East respiratory syndrome (MERS), severe acute respiratory syndrome (SARS), Ebola, Zika, and Dengue [1], while advancing the theory of Disease X, a disease with an epidemic or pandemic risk that is caused by a still unknown pathogen [2]. The recent outbreak at the end of 2019 of severe pneumonia with an uncertain etiopathology in China was initially indicated as the Disease X by WHO. Only after the identification of the pathogen as a new coronavirus (CoV), this new disease has been renamed COVID-19 [3,4]. The new CoV is called SARS-CoV-2 due to its high similarity to SARS-CoV (79.5% genome identity), as revealed by genomic sequencing. The infection has rapidly spread worldwide causing the pandemic COVID-19 disease, as announced by WHO on 11 March 2020, which has currently caused more than seven million cases, many hospitalization, and about 400,000 deaths [5]. The lack of vaccines or therapies against the virus led to globally consider the social distancing the only option to slow down the spread of the infection, indeed it work very well in some country [6,7,8].

Nowadays, drug repurposing represents a valid strategy for redeveloping existing approved or investigational drugs for new therapeutic purposes enabling the fast introduction into clinical setting [9]. For instance, “Solidarity” is an international clinical trial to help finding effective COVID-19 treatments, launched by WHO [10]. Among the drugs in trials, Remdesivir is an intravenous monophosphoramidate nucleoside prodrug, originally under investigation as Ebola polymerase inhibitors. Remdesivir received emergency use authorization in US, Japan, and India for the treatment of patients with severe manifestations of COVID-19. The authorization was granted on the basis of the promising results from the National Institute of Allergy and Infectious Diseases (NIAID) and Gilead clinical trials, and from the compassionate use programs [11,12].

However, the most effective antiviral regimens, such as those for the management of HIV or the cure of HCV infections, are based on the combination of at least two drugs with different mode of action, thus ensuring higher efficacy and resistance barrier with respect to a single agent also shortening therapy to achieve the sustained virologic response (SVR). When considering the worldwide emergency of COVID-19 disease, the entire scientific community cooperated to characterize the virus morphology and replication steps in order to identify a vaccine and/or a cure [13]. Many efforts have been made in molecular biology, biochemistry, and structural biology of SARS-CoV-2. The rapid sharing of these data together with the pre-existing knowledge about parent CoVs, especially SARS-CoV and MERS-CoV, are a valuable starting point for the discovery of broad-spectrum CoV inhibitors to potentially protect from emerging infection that is caused by members of the same virus family [13].

**Figure 1:**
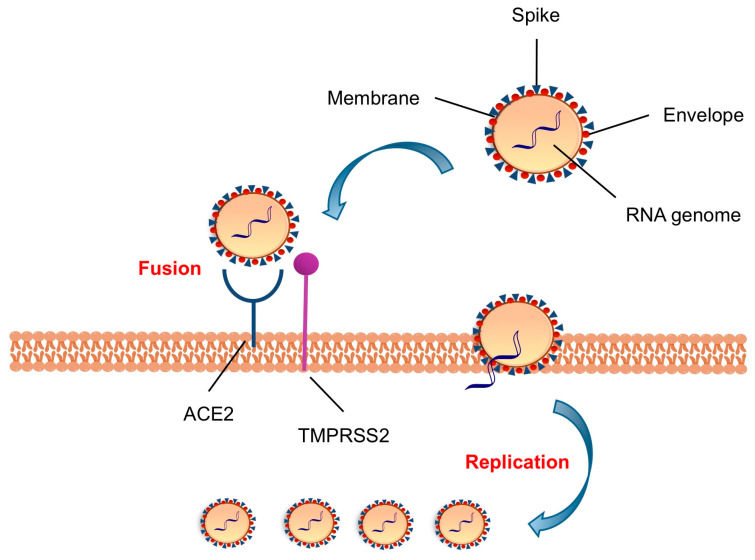
Schematic representation of SARS-CoV-2 virion and viral entry into host cell.

CoVs belong to one of two subfamilies of *Coronaviridae*, namely *Coronavirinae*, which is, in turn, subdivided into four genera (α, β, γ, and δ) based on the different phylogenetic clustering, and each genus further comprises various subgroups [14]. Several CoVs were already known to be human pathogens, such as α-CoVs, i.e., HCoV-229E and HCoV-NL63, and β-CoVs, such as SARS-CoV, MERS-CoV, HCoV-OC43, HCoV-HKU1, and the new SARS-CoV-2 [14]. While β-CoVs HCoV-OC43 and HCoV-HKU1 are associated with causing common cold and minor symptoms, SARS-CoV and MERS-CoV, as well as the most recent SARS-CoV-2, penetrate in the lower respiratory tract, causing serious complications. The infection can spread towards other organs, thus making the clinical picture worse, especially the heart, kidney, liver, intestine and central nervous system. α- and β-CoVs are very common among bats and rodents [14]. Among human CoVs, SARS-CoV is the closest to SARS-CoV-2 with 79% genetic similarity [15], but the highest genetic similarity is shared with bat coronavirus RaTG13 (98%) [16], as a demonstration of possible interspecies spread, known as spill-over [17]. Although a lower mortality rate has been estimated until now, the higher transmissibility of SARS-CoV-2 over SARS-CoV is a determinant factor of its pathogenic danger [18]. CoVs share morphological, structural, and functional features, making a relevant contribution in knowledge of SARS-CoV-2 infection, pathogenesis, and treatment options [4,17]. CoVs are enveloped viruses with a large diameter (50–200 nm) and contain a non-segmented, positive-sense single stranded (ss-(+))-RNA genome of ~30 kb, which is the largest identified viral RNA genome to date [19]. Similar to other CoVs, SARS-CoV-2 appears in a spherical shape by cryo-electron microscopy (cryo-EM), with Spike (S) glycoproteins that emanate from virion surface and give the characteristic crown-like appearance (Figure 1) [19]. The S protein constitutes the viral envelope together with other two structural proteins, namely envelope (E) and membrane (M), while the nucleocapsid (N) protein, inside the envelope, binds and protects the RNA genome. 

The SARS-CoV-2 S glycoprotein drives tissue tropism and mediates attachment to the host cell through the interaction between the receptor binding domain (RBD) within the S1 region and the human angiotensin-converting enzyme 2 (ACE2) receptor, the same host receptor that mediates the interaction of SARS-CoV [20,21]. The major degree of infectivity that characterizes SARS-CoV-2 over SARS-CoV has been appointed to an estimated 20-fold higher binding affinity of the SARS-CoV-2 RBD toward ACE2 [20]. Following receptor binding, the access to the host cellular cytosol is realized by proteolytic cleavage of the S protein at the S1/S2 and S2’ sites, due to the transmembrane protease serine 2 (TMPRSS2) and/or a cathepsin B/L (CatB/L) [21]. The cleavage at site S2’ exhibits a fusion peptide that inserts into the host cell membrane and leads to the formation of an antiparallel six-helix bundle (6-HB), allowing the fusion process and, therefore, the uncoating and release of the viral RNA into the cytoplasm [22]. The next step in the CoV lifecycle is the translation of the replicase gene from the viral RNA into polyproteins pp1a and pp1ab that afford non-structural proteins thanks to autocleavage mediated by viral main protease and papain-like protease [23]. The non-structural proteins form the replicase–transcriptase complex for the viral genome replication. The genome also encodes structural proteins (S, E, M, and N), which assemble into new virions during the late stage of viral life cycle, and accessory proteins [19], associated to viral pathogenesis and host immunity suppression [24].

Researchers all around the World are exploiting different strategies that target both viral and host factors that are essential for viral replication, thus producing a great amount of data making COVID-19 drug discovery an emerging, rapidly evolving, and intriguing research field. Besides targeting intracellular steps of viral replication, virus entry into host cells represent another way to prevent/treat viral infections. Two drugs targeting HIV-1 entry are currently used in antiviral retro-therapy, the small molecule chemokine receptor antagonist Maraviroc [25], which binds to CCR5 and prevents interaction with HIV gp120, and Enfurtivide [26], a peptide fusion inhibitor that is able to bind to gp41 blocking its fusion activity with host cell membrane. Moreover, many inhibitors of viral entry are in different phases of development for different enveloped viruses, for instance, Fostemavir is a blocker of CD4 in clinical development [27]. Therefore, inhibiting the entry step is a validated strategy for the prevention/treatment of viral infection [28]. The CoV S fusion protein and the S/ACE2 interaction are viral targets to inhibit entry; indeed, a number of inhibitors have been described for SARS-CoV-2 and/or parent CoVs. Moreover, the cellular TMPRSS2 and CatB/L proteases represent potential host factors to identify other classes of entry inhibitors. In this review, small molecules and peptides targeting S will be analysed, while biologicals are outside the scope of this contribution. Additionally, the most promising host targeting strategies, involving TMPRSS2 and CatB/L exploited against CoVs and other viruses, will be also reviewed.

## 2. Targeting S Protein to Block Viral Entry 

The attachment of virus particles to cell-surface receptors is the first common step between both non-enveloped and enveloped viruses, which represent the earliest stage of infection allowing the viruses to enter the cells through endocytic and non-endocytic pathway. In most cases, the process is regulated by several glycosylated membrane proteins that allow for the recognition of host target cells, acting as anchors. In the known human CoVs (i.e., 229E, NL63, OC43, HKU1, MERS-CoV, SARS-CoV, SARS-CoV-2), the S glycoproteins mediate attachment to host cell surface and entry. The S protein is a 1200 aa long homotrimeric class I fusion protein, synthesized in the secretory pathway of the host cells, comprising three segments: a large ectodomain with a receptor-binding subunit S1 and a membrane-fusion subunit S2, a single-pass transmembrane anchor, and a short intracellular tail [22]. The N-linked glycans protruding from the trimer surface are responsible for the S folding and also for the recognition by neutralizing antibodies (Abs) [29]. 

Among CoVs, the S1 subunits show little sequence similarity having markedly different RBDs. However, SARS-CoV-2 enters into cells, like SARS-CoV, through the binding of RBD to the host ACE2 protein in cells from different species (human, bats, civets, pigs, and mice receptors) [16]. Conversely, the S2 domain is highly conserved and contains the fusion peptide (FP), heptad repeat 1 (HR1), heptad repeat 2 (HR2), transmembrane region (TM), and cytoplasmatic tail (CT). As a result, the SARS-CoV-2 S protein shows about 76% amino acid sequence identity to SARS-CoV S, with RBD less conserved (64% identity) than the S2 fusion domain (90% identity) [20]. Indeed, SARS-CoV and SARS-CoV-2 have highly similar HR1 and HR2 regions with identity of 92.6% and 100%, respectively [20]. 

**Figure 2:**
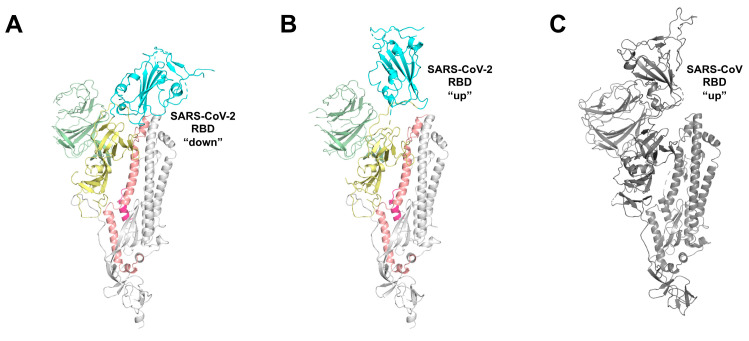
Receptor binding domain (RBD) “down” and “up” conformations of SARS-CoV-2. (**A**) Single protomer of SARS-CoV-2 with the RBD in the down conformation, displayed as cartoon (PDB ID: 6VSB). RBD is colored cyan, the N-terminal domain (NTD) is pale green, subdomains 1 and 2 (SD1 and SD2) are yellow, the S2 domain is white, with HR1 colored salmon and FP hotpink. (**B**) Single protomer of SARS-CoV-2 in the RBD up conformation next to (**C**) a protomer of SARS-CoV (colored gray) in the RBD up conformation (PDB: 6CRZ).

Cryo–electron microscopy structures of SARS-CoV (PDB IDs: 3JCL [30], 5X58 [31]) and SARS-CoV-2 (PDB IDs: 6VXX [32], 6VSB [20]) S-trimer revealed a metastable prefusion conformation inaccessible to host receptor (“down” conformation), which changes in a less stable receptor-accessible state (“up” conformation), exposing the determinants for receptor binding (Figure 2). 

The RBD undergoes a hinge-like movement to bind the peptidase (PD) domain of ACE2, resulting in a shedding in S1 and a cleavage in S2’ site, with a refolding in S2 to adopt a stable post-fusion hairpin conformation [33,34]. This six-helix bundle (6-HB) drives viral and cell membranes in close proximity for the fusion event [20]. 

**Figure 3:**
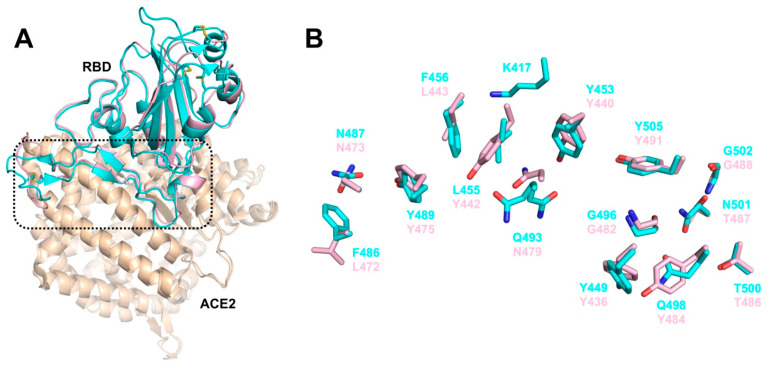
Structural details of the interface between SARS-CoV and SARS-CoV-2 RBDs and ACE2. (**A**) Structural overlay of SARS-CoV-2 (cyan, PDB ID: 6M0J) and SARS-CoV (light pink, PDB ID: 2AJF) RBDs bound to ACE2 (wheat), displayed as cartoon. The four disulfide bonds in SARS-CoV-2 RBD are shown as sticks. The region enclosed by the black dashed lines, encompassing the interface between RBD and ACE2 is illustrated in detail in panel (**B**). Overlay of the RBD interface residues of SARS-CoV-2 (cyan sticks) and SARS-CoV (light pink sticks). Q493 is shown in two alternate positions.

**Figure 4:**
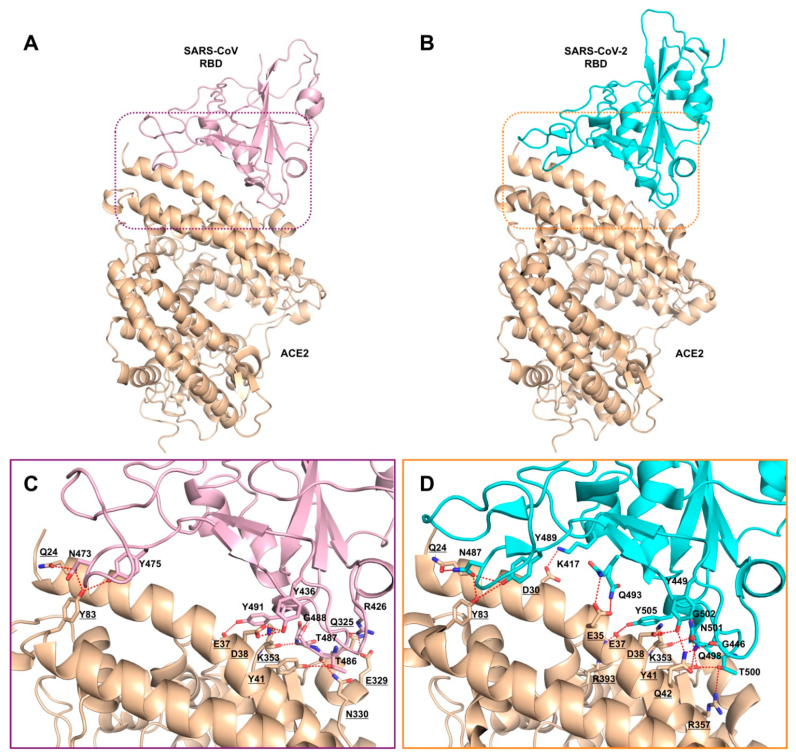
Comparisons of the interactions formed at the interfaces between SARS-CoV and SARS-CoV-2 RBDs and ACE2. Overall structure of (**A**) SARS-CoV (light pink, PDB ID: 2AJF) and (**B**) SARS-CoV-2 (cyan, PDB ID: 6M0J) RBDs bound to ACE2 (wheat). Key residues involved in SARS-CoV RBD/ACE2 (**C**) and SARS-CoV-2/ACE2 (**D**) complex formation are shown as sticks and labeled. ACE2 residues are underlined for clarity. Hydrogen bonds and salt bridges are displayed as dashed red lines.

The X-ray crystal structure of SARS-CoV RBD domain (residues 306–527) in complex with ACE2 (residues 19 to 615) (PDB ID: 2AJF) provides structural basis for understanding features of SARS-CoV S protein involved in receptor binding [35]. The S protein discloses a core structure that formed by five-stranded antiparallel β-sheets and an RBD presenting a loop-dominated concave surface with at the basis two-stranded antiparallel β-sheets and two ridges formed by loops interactions [35]. The RBD needs Tyr442, Leu472, Asn479, Thr487, and Tyr491 to bind ACE2 and shows a gently concave surface holding the N-terminal lobe of the peptidase domain of ACE2 [35]. The three-dimensional (3D)-structural model of SARS-CoV-2 S protein, constructed through homology modelling with SARS-CoV S protein (PDB ID: 6ACD) [36], shows almost identical structure in RBD domains [37]. Through structural superimposition and molecular rigid docking approaches, a 3D model of SARS-CoV-2 RBD motif complexed with ACE2 has been described, showing similar Van der Waals and electrostatic properties in the interaction interface with ACE2 [37]. Structural features for the recognition of the SARS-CoV-2 by the full-length human ACE2 have been reported, pointing to concurrent binding of two S protein trimers to dimeric ACE2. Essential amino acids for interactions between SARS-CoV-2 RBD and ACE2 and differences of interfaces between SARS-CoV RBD and SARS-CoV-2 RBD have been definitely elucidated through solving the X-ray crystal structure of the SARS-CoV-2/ACE2 complex (PDB ID: 6M0J) [38]. In particular, α1-helix of protease domain of ACE2 shows the main polar interactions with RBD, while α2-helix and the linker between β3 and β4 contribute partially to interaction. RBD motif contains nine cysteines residues with eight of them forming four disulfide bridges, in particular three in RBD core and one in distal loops. Eight identical amino acids (Tyr449/Tyr436, Tyr453/Tyr440, Asn487/Asn473, Tyr489/Tyr475, Gly496/Gly482, Thr500/Thr486, Gly502/Gly488, and Tyr505/Tyr491) have been found in SARS-CoV-2 and SARS-CoV RBDs, respectively. On the other hand, key amino acids of SARS-CoV RDB Tyr442, Leu472, Asn479, and Thr487 are replaced by Leu455, Phe486, Gln493, and Asn 501 in SARS-CoV-2 RDB, which establish more interactions with the host receptor, along with unique salt bridge between S Lys417 and ACE2 Asp30. Altogether, these differences may explain the approximately 20-fold higher affinity of SARS-CoV-2 S to ACE2 with respect to SARS-CoV and are likely at the base of higher transmissibility of COVID-19 infection (Figure 3 and Figure 4) [38,39]. 

A focus on S1/S2 and S2’ cleavage sites confirm a strictly conserved S2’ site and fusion peptide among SARS-CoV-2, SARS-CoV, and bat-CoV (SL-CoV-RaTG13), while S1/S2 site contains a 681PRRA684 sequence and a conserved Arg685 in fifteen SL-CoV-RaTG13 sequences analysed [40]. These three arginines are recognized and proteolytically processed by the transmembrane protease TMPRSS2, which is highly expressed in the respiratory epithelium cells. 

**Figure 5:**
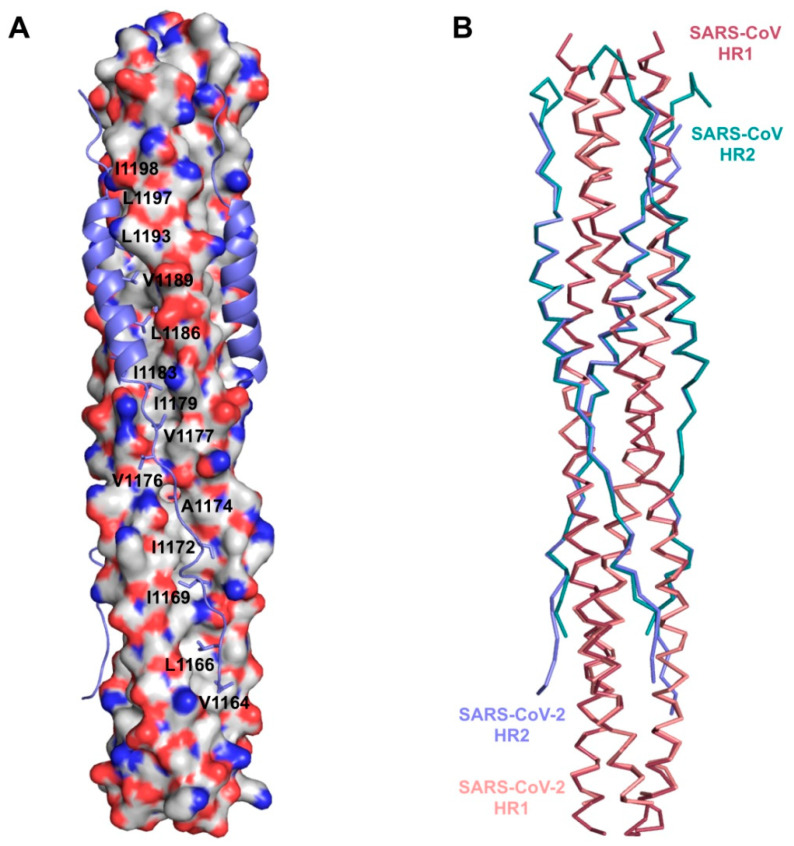
Structure of 6-HB fusion core in SARS-CoV-2. (**A**) The HR1 domain of SARS-CoV-2 is depicted as electrostatic surface, with hydrophobic residues in white, basic in blue, and acidic in red. The HR2 domain is shown in cartoon representation, with the hydrophobic residues in the central fusion core region shown as sticks and labeled. (**B**) The superposition of 6-HB structures of SARS-CoV-2 (PDB ID: 6LXT) and SARS-CoV (PDB ID: 1WYY), shown as ribbon. The HR1 and HR2 domains are colored salmon and slate for SARS-CoV-2, raspberry, and deep teal for SARS-CoV, respectively.

A comparison between the 6-HB fusion core structure of SARS-CoV-2 and SARS-CoV S proteins, in order to investigate the structural basis for S-mediated membrane fusion process, has been carried out analysing recombinant fusion proteins containing the major parts of HR1 (residues 910–988) and HR2 (residues 1162–1206) merged with a linker (L6, SGGRGG). When comparing the X-ray crystal structures of post fusion hairpin conformation of SARS-CoV S2 (PDB ID: 1WYY) [41] and the recently solved post fusion core of SARS-CoV-2 S2 subunit (PDB ID: 6LXT) [42], the overall 6-HB structure of SARS-CoV-2 appears to be highly conserved with respect to those of other HCoVs, including SARS- and MERS-CoVs, presenting hydrophobic residues (Val1164, Leu1166, Ile1169, Ile1172, Ala1174, Val1176, Val1177, Ile1179, Ile1183, Leu1186, Val1189, Leu1193, Leu1197, and Ile1198) in the central fusion core region (Figure 5).

**Figure 6:**
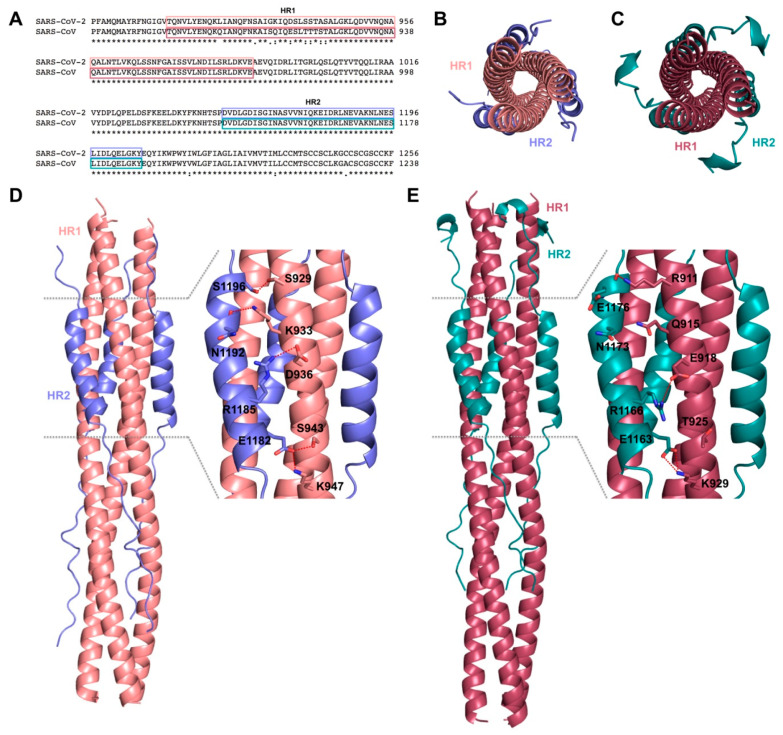
Comparisons of 6-HB fusion core in SARS-CoV-2 and SARS-CoV. (**A**) Sequence alignment between SARS-CoV-2 and SARS-CoV S proteins for the HR1 and HR2 regions, indicated with boxes colored salmon and raspberry for HR1 and slate and deepteal for HR2, respectively. Top view of the 6-HB fusion core structure of (**B**) SARS-CoV-2 (PDB ID: 6LXT) and (**C**) SARS-CoV (PDB ID: 1WYY) displayed as cartoon. The HR1 and HR2 domains are colored as in (**A**) and labeled. Side view of 6-HB of (**D**) SARS-CoV-2 and (**E**) SARS-CoV. A zoomed view of the interactions between HR1 and HR2, mediating fusion core formation, is shown on the right side. Key residues are displayed as sticks and labeled; hydrogen bonds and salt bridges are displayed as dashed red lines.

The fusion core of the SARS-CoV-2 S2 reveals eight different aminoacids in HR1 domain that provide stronger interactions between HR1 and HR2, whereas the HR2 domain results fully identical; in particular, Ser929, Arg933, Asp936, Ser943, and Lys947 provide new strong H-bonds and stronger salt-bridge interactions, explaining the higher fusion activity when compared to SARS-CoV (Figure 6) [42].

Therefore, the considerable amount of structural data on SARS CoV-2-RBD/ACE2 interaction, revealing key amino acid residues at the contact interface between the two proteins, provides useful structural information for driving the development of disruptors of the SARS-CoV-2/ACE2 interaction. Rather than small molecules, peptides represent accessible inhibitors that are able to efficiently cover an extended surface of protein-protein interaction containing many key hot spots. Thus, peptides targeting S protein offer strategies for the development of specific viral entry inhibitors. 

Based on the 3D structure of SARS-CoV-2 RDB/ACE2 (PDB ID: 6M17), a computational campaign that is based on molecular dynamic simulations of peptides extracted from the α1-helix of ACE2 led to the identification of 23-mer peptide SBP1 (IEEQAKTFLDKFNHEAEDLFYQS), corresponding to the N-terminal ACE2 α1 helix [43]. The 23-mer peptide SBP1 can likely stably bind to SARS-CoV-2 RBD with a *K*_D_ of 47 nM (as measured by biolayer interferometry), which is comparable to full length ACE2 binding affinity to SARS-CoV-2 RBD (14.7 nM) [20]. Differently, SPB2, a truncated analogue in the middle of SPB1 peptide, is not able to associate with the S-RBD. Authors assess that further chemical modifications of SPB1 amino acids sequence are ongoing in order to increase the binding affinity to SARS-CoV-2 RBD and produce peptide potentially proteolytically resistant.

However, when considering the high variability of RBD regions within S protein across different CoVs, the RBD motif results not an ideal target for the design of broad-spectrum inhibitors. Otherwise, HR1 and HR2 conserve their sequences among CoVs and play a key role in viral fusion by forming the 6-HB [44]. Thus, the identification of peptide fusion inhibitors may represent a more advantageous strategy to fight CoVs infection. Based on X-ray crystal structures of MERS- and SARS-CoVs S protein fusion cores, several peptides derived from HR1 and HR2 sequences have been developed [42,44]. Unfortunately, SARS- and MERS-CoVs HR1 peptides do not display antiviral activity likely due to their tendency of aggregation in the absence of HR2 peptides and the inability of forming trimer structures. In contrast, several peptides derived from the HR2 region that could bind the HR1 sequences have been previously reported for the inhibition of enveloped viruses [45,46,47,48]. For instance, Enfuvirtide is the first 36-amino acids fusion inhibitor, derived from the HR2 domain of gp41, approved as drug for the treatment of HIV [26], as well as various peptides are currently different phase of development for the treatment of other viruses infections [49,50,51].

**Figure 7:**
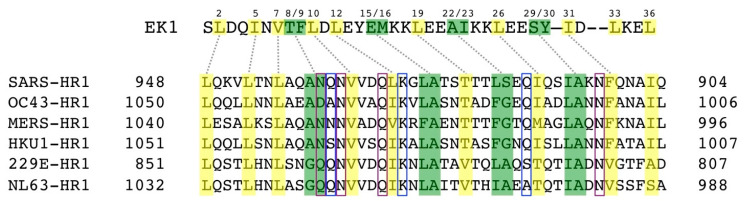
The interactions of the broad-spectrum peptide inhibitor EK1 with HR1 residues of different HCoVs. EK1 and HR1 residues connected with dashed gray lines locate to the same layers on the 3HR1 triple helix. Burying EK1 residues are highlighted in yellow, and ridge-packing EK1 residues are highlighted in green. HR1 residues that mediate assembly of the 3HR1 cores are highlighted in yellow, while those involved in ridge packing are highlighted in green. HR1 residues forming conserved side chain-to-side chain and side chain-to-main chain hydrophilic interactions with EK1 residues are indicated with boxes colored blue and purple, respectively. Adapted from ref. [46].

Peptides derived from HR2 regions of SARS-CoV [48] and MERS-CoV [47] have been previously reported as able to inhibit HCoV S-mediated cell-cell fusion, using effector cells carrying S-GFP fluorescent fusion protein. HR2P (SLTQINTTLLDLTYEMLSLQQVVKALNESYIDLKEL) of MERS-CoV shows an IC_50_ = 0.6 µM in the cell fusion assays, while two HR2P mutants, HR2P-M1 and HR2P-M2, with two and seven mutations, respectively, able to form intramolecular salt-bridges, have shown higher stability and water-solubility than their precursor HR2P peptide retaining a similar potency [47]. Likewise, the CP-1 peptide (GINASVVNIQKEIDRLNEVAKNLNESLIDLQELGKYE) of SARS CoV HR2 domain shows an IC_50_ = 19 µM in the cell fusion assay [48]. Unfortunately, these HR2 derived peptides do not cross-inhibit MERS-CoV and SARS-CoV, lacking broad-spectrum antiviral activity against heterologous HCoVs. The insertion of some charged amino acids like Glu and Lys, as reported for HIV-1 fusion inhibitor peptides, increases the solubility and stability of these peptides as well as the introduction of mutations at specific sites not involved in HR1 binding, enhances the antiviral activity [52]. By applying the same strategy to the HR2P peptide derived from the short 6-HB fusion core of OC43-CoV, EK1 has been obtained (Figure 7). EK1 peptide shows a broad-spectrum inhibition with IC_50_ ranging from 0.19 to 0.62 µM in the cell fusion assays for MERS-, SARS-, 229E-, NL63-, OC43-, Rs3367-, WIV1-, and SHC014-CoVs [46]. EK1 displayed inhibitory activity in pseudovirus infection (PsV) assays against MERS-CoV (IC_50_ = 0.26 μM), SARS-CoV (IC_50_ = 2.23 μM, 229E (IC_50_ = 3.35 μM), NL63 (IC_50_ = 6.02 μM), and OC43 (IC_50_ = 1.81 μM), Rs3367 (IC_50_ = 2.25 μM) and WIV1 (IC_50_ = 2.10 μM) [46]. Evaluation against live HCoV infections of MERS-, OC43-, 229E-, and NL63-CoVs has shown that EK1 inhibits the infection and replication in a dose-dependent manner. Moreover, the intranasal administration of EK1 protects mice infected with OC43 and MERS-CoV infection, increasing the survival rate [46]. Preliminary data showed that the EK1 peptide is effective against SARS-CoV-2 S protein mediated membrane fusion and PsV infection in a dose-dependent manner [44].

**Figure 8:**
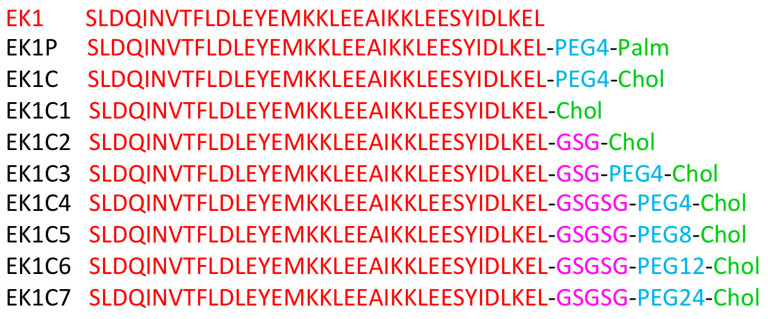
Amino acids sequences of EK-1 and its lipopeptide derivatives.

The lipidation strategy has been reported for various fusion peptides inhibitors as an advantageous modification to improve activity and in vivo properties (e.g., LP-19 peptide of HIV-1) [53,54]. Similarly, the lipidation strategy was also undertaken for optimization of EK-1 peptide which was derivatized with cholesterol and palmitic acid covalently bound to the C-terminus with a spacer of polyethylene glycol, yielding lipopeptides EK1C and EK1P with IC_50_ = 48.1 nM and 69.2 nM, respectively, in the cell fusion assay using SARS-CoV-2 S [42]. Seven cholesterol-EK1 adducts having different linkers, based on glycine/serine sequence (GSG) and/or a PEG- based spacer (Figure 8). In particular, by extending the GSG linker to a longer GSGSG as in EK1C4 peptide increase potency with IC_50_ = 1.3 nM in the cell-cell fusion assay, suggesting that the linker length could play a crucial role in lipopeptides. Conversely, an increase in PEG-based linker length (e.g., EK1C5, EK1C6 and EK1C7) produces a slight reduction of cell-cell fusion inhibition with IC_50_ = 3.1, 3,9, and 3.9 nM, respectively. Thermal shift experiments confirmed the specific binding of EK1C4 to S-HR1s of SARS-CoV-2 and SARS- and MERS-CoVs. Moreover, one possible model of interaction suggests that the portions of EK-1 and cholesterol in EK1C4 could bind to two of the three hydrophobic grooves of HR1, but still the real structural information need to be elucidated. Interestingly, EK1C4 possesses the most potent activity in cell-based infection assays by live CoVs, such as SARS-CoV-2, MERS-CoV, HCoV-OC43, HCoV-229E, and HCoV-NL63 (EC_50_s ≤ 180 nM), with low cell toxicity (CC_50_ > 5 µM, SIs > 136). In particular, EK1C4 potently inhibits SARS-CoV-2 replication, with EC_50_ = 36.5 nM, resulting 67-fold more potent than EK1 (EC_50_ = 2.47 µM) in the same assay. More importantly, EK1C4 tested in mice that were infected with HCoV-OC43, by intranasal administration of the lipopeptide (0.5 mg/kg) at different time point pre- (0.5, 2 h, 4 h, 12, and 24 h) and post- (0.5 and 2 h) infection. Pre-treatment from 0.5 to 4 h resulted in the 100% survival rate, at lower dosage than parent EK1 (20 mg/kg), while the protecting effect decreased to 83 and 0% at 12 and 24 h, respectively. Post-treatment at 0.5 h was highly effective (100%) while after 2 h the effect was low (17%).

**Figure 9:**
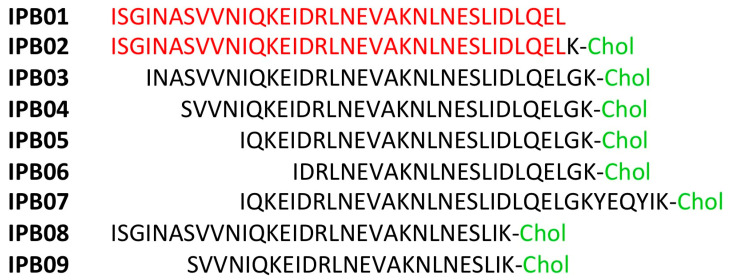
SARS-CoV-2 HR2-derived peptides.

Analogously, other HR2 sequence-based fusion inhibitors have been reported as C-term cholesterol-linked IPB01 lipopeptides (Figure 9) [55]. IPB01-IPB04 peptides potently inhibit cell fusion in a dual-split protein (DSP)-based cell fusion assay on SARS CoV-2, showing IC_50_ between 15 and 33 nM. However, only IPB02 maintained comparable activity in cell-based assays against SARS-CoV-2 PV with EC_50_ = 0.08 μΜ, while truncation at the N-terminus as in IPB03 and IPB04 resulted in 12- and three-fold reduction in activity, respectively. IPB01 lacking the cholesterol unit is instead only weak active (EC_50_ = 33.7 μΜ) in cell-based assay, thus lipidation strategy is very important to improve activity in the cell based assay. A further N-terminal truncation results in inactive peptides (IPB05, IPB06) in both assays. On the other hand, N-truncated IPB07 having six amino acids of membrane proximal external sequence at C-terminus partially restores its activity, showing a similar profile to IPB03, while the only deletion of some aminoacids at the same C- terminus, as in IPB08, leads to weak inhibitor with low μM potency in both assays. Finally, IPB09 shows that both N- and C-terminus are crucial for the inhibitory activity, as this truncated peptide lost activity. These fusion peptides inhibitors are specific for CoVs, as that they are inactive (IC_50_ > 50 μM) when tested against Vescicular stomatitis virus pseudoviruses used as a control.

Recently, Nelfinavir mesylate (Viracept), an anti-HIV protease inhibitor acting also as anticancer agent inducing apoptosis and necrosis, and also able to inhibit SARS-CoV replication [56], has been reported as an inhibitor of SARS-CoV-2 S-mediated cell-cell fusion, thus blocking the formation of multinucleated cells [57]. Syncytia formation is an important cytopathic phenomenon because the virus can spread from cell-to-cell avoiding extracellular spaces and exposure to neutralizing antibodies. Moreover, virus-induced cell fusion within CoV infection can increase an inflammatory response leading to adverse effects in the host. In immunofluorescence experiments, Nelfinavir has been shown to act on both SARS-CoV-2 and SARS-CoV S glycoproteins-mediated cell-cell fusion with the complete inhibition at 10 μM without affecting the S cell surface expression. Computational analysis suggests that nelfinavir could bind to the trimeric S, close to the fusogenic domain, but there is no experimental validation. Based on pleiotropic effect previously reported for nelfinavir on ER stress, the authors speculate that the compound could interfere with post-translational processes, or probably could block cellular proteases responsible for S-n or S-o fusion activation, but also these hypotheses are not supported by experimental evidences.

## 3. Targeting Host Protease to Block Viral Entry

The proteolytic cleavage of S protein at the S1/S2 and S2′ sites by the serine protease TMPRRS2 and/or endosomal cysteine proteases CatB/L drives viral entry through the fusion peptide that inserts into the host cell membrane. This insertion leads to the formation of an antiparallel six-helix bundle, allowing the fusion process and, therefore, the uncoating and release of the viral RNA in the cytoplasm. The S1/S2 and S2′ priming events by host proteases are necessary for SARS-CoV-2 to infect the host and interfering with the virus entry may turn out to be an advantageous antiviral strategy, as it would block the infection or virus propagation at an early stage, more importantly if considering its high transmissibility. Targeting host factors has the advantage of reducing the possible development of drug resistance and of likely providing broad-spectrum activity, by contrast interacting with the host protein, the possibility to have more severe side effects are higher with respect the classical antiviral approach. However, TMPRRS2 and CatB/L involvement in viral infection is still the object of study and targeting these host proteases for CoV treatment is an emerging strategy. In addition, the available data suggest that simultaneous inhibition of both proteases is required for robust block of antiviral entry [58,59]. So far, there are no reports of medicinal chemistry programmes focusing of TMPRRS2 and cathepsin B/L within CoV drug discovery, thus available inhibitors often show limitations, in term of potency, selectivity, drug-like properties. Nevertheless, some compounds have been shown to exert a promising antiviral effect against SARS-CoV-2 and/or other related CoVs and are described below.

### 3.1. TMPRSS2 as Host Target and Its Inhibitors

**Figure 10:**
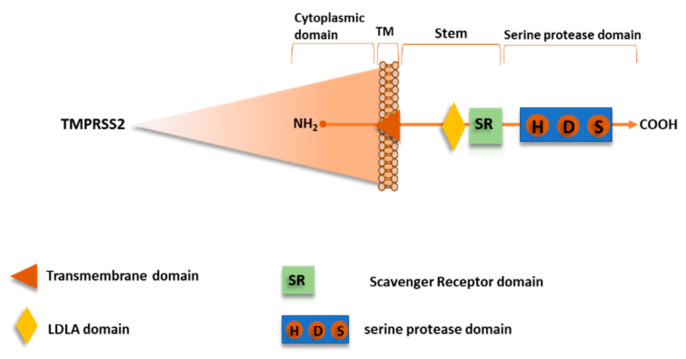
Schematic representation of serine protease TMPRSS2.

Recently, TMPRSS2 has been shown to mediate SARS-CoV-2 S protein priming, as well as for SARS-CoV and other CoVs [21,58,60,61,62]. TMPRSS2, also named Epitheliasin, is a 492 aa serine protease of type II transmembrane serine proteases (TTSPs) family, expressed on the cell surface, consistently to their role in regulating cell-cell and cell-matrix interactions. The human TTSP family includes 17 members so far sharing the same structural features. The *N*-terminal intracellular domain preserves the phosphorylation sites, followed by the transmembrane domain, and the stem region that is located in the initial extracellular part with a binding site for low-density lipoprotein (LDL) and calcium in a LDL Receptor A motif and a single scavenger receptor Cys-rich (SRCR domain). The *C*-terminal extracellular endoprotease domain contains the catalytic triad composed by the residues His-Asp-Ser, in which the Ser hydroxyl group promotes nucleophilic attack on the priming site (Figure 10) [63]. 

TMPRSS2 is predominantly expressed in prostate, but it has also been found in lungs, colon, liver, kidneys, and pancreas. The expression in the upper airways, bronchi and lungs, where its physiological function remains unclear, gives thought to its important role for pneumotropism of several highly pathogenic viruses, such as SARS-CoV-2, SARS-CoV, MERS-CoV, and HCoV-NL63. Indeed, CoVs engage ACE2 to enter into host cells and although the ACE2 is a ubiquitous enzyme, they show particular tropism for the lungs. In addition, while ACE2 is expressed in both type I and type II pneumocytes, it has been verified that SARS-CoV readily infects the type I pneumocytes at early stage [60,64]. In vivo experiments showed that TMPRSS2 is responsible of viral spread and immunopathology of CoVs infection [59]. In TMPRSS2_ko mice, SARS-CoV and MERS-CoV showed significantly reduced viral replication in the lungs, especially in the bronchioles, and the inflammatory infiltration. Moreover, TMPRSS2 is not only involved with CoVs S protein activation, but its role has also been recognized in the activation of glycoprotein on the surface of influenza A virus, metapneumovirus, and porcine epidemic diarrhoea virus in different stages of viral life cycles [65]. Therefore, targeting TMPRSS2 could be a broad-spectrum antiviral strategy; however, neither drugs able to specifically inhibit this target have been identified, nor sufficient information about the substrate specificity and no 3D structures of the protein are available. However, it was reported that the fluorogenic trypsin substrates Cbz-Gly-Gly-Arg-AMC [66] and Boc-Leu-Gly-Arg-AMC [67] were also substrates for TMPRSS2, indicating that P1 can be represented by Arg, which is consistent with the recognition elements of some drugs able to inhibit human epithelia serine proteases; anyway enzymatic kinetics analyses were not performed and values of Km or Kcat are not known [67]. On the other hand, drugs that are able to inhibit a wide panel of human serine proteases, including TMPRSS2, are currently approved to treat prostate cancers and several inflammatory pathologies [68]. 

**Figure 11:**
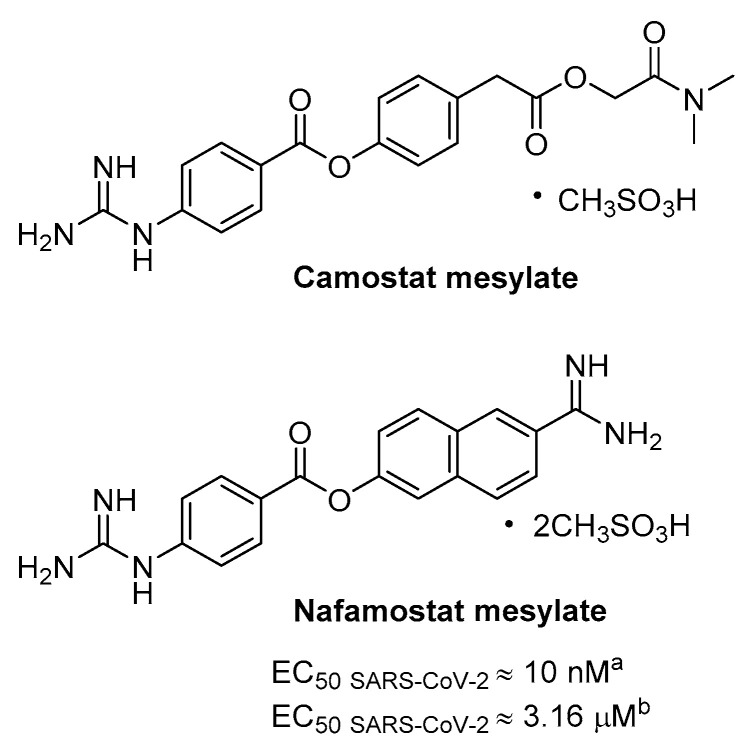
Chemical structures of phenyl-4-guanidinobenzoate derivatives known drugs as serine protease and antiviral activity of nafamostat agaisnt SARS-CoV-2 in Calu-3 cells measured by CPE; ^a^ pre-treatment; ^b^ added after virus inoculation.

Previous evidences showed that the clinically proven serine protease inhibitor camostat mesylate was able, even at high concentration (up to 100 µM), to partially block SARS-CoV infection (65% of inhibition) in cell expressing TMPRSS2 without causing toxicity; hence, the antiviral activity was enhanced by adding a CatB/L inhibitor, namely E64d, thus indicating that the remaining 35% was attributable to endosomal cathepsins [58]. Camostat is a pseudo irreversible inhibitor of different serine protease, including TMPRSS2, being characterized by aromatic guanidine as P1 mimetic recognition elements (Figure 11), less polar than Arg, and it is used in Japan for prostate cancer and other applications, such as pancreatitis and liver fibrosis. Consistently, camostat produced a partial block (50–60%) of SARS-CoV-2 entry in TMPRSS2+ cell lines, including Calu-3 lung cells, while no effect was observed in TMPRSS2- cells, and full inhibition was obtained again adding E64d [21]. Interestingly, animal model studies have found that the treatment of camostat mesylate not only produced a 10-fold reduction SARS-CoV titers in Calu-3 airway epithelial cells [69], but also an increase of survival rate of 60% in mice [70]. Camostat can inhibit in vivo infection by SARS-CoV and other pneumoviruses known to utilize TMPRSS2; therefore, the drug could be a suitable antiviral candidate for drug repurposing as component of a drug combination, to prevent infections in the lungs by SARS-CoV-2. Indeed, camostat has recently been involved in an interventional study to evaluate the efficacy and safety in humans of inhibiting SARS-CoV-2 infection, which provides a randomized treatment in 580 participants with camostat mesylate drug (Phase I) and in parallel with placebo oral tablet (Phase IIa) (ClinicalTrials.gov Identifier: NCT04321096). In order to obtain the highest grade of evidence, double-blinded, randomised, placebo controlled trials are carried out on 334 patients with moderate COVID-19 infection. The clinical trial is in phase IV, but not yet recruiting (ClinicalTrials.gov Identifier: NCT04338906).

Nafamostat is a serine protease inhibitor that is structurally related to camostat, in use as anticoagulant used for disseminated intravascular coagulation (DIC), that has proven particularly potent activity in blocking CoV infection in vitro likely by inhibiting TMPRSS2 mediated entry (Figure 11). A dual split protein (DSP) reporter assay was developed to quickly monitor membrane fusion mediated by viral S protein and to screen a library of approved drugs, leading to the identification of nafamostat as a potent inhibitor of fusion S activity of MERS. Tested in MERS infection assay the compound blocked viral replication by 100-fold at a very low concentration of 1 nM, more efficiently than camostat [71]. More recently, the same research group has exploited the DSP using SARS-CoV-2 S protein where nafamostat shows fusion inhibitory activity and more interesting the drug inhibits with excellent potency SARS-CoV-2 replication in pulmonary Calu-3 cells with EC_50 CPE_ of about 10 nM with pre-treatment [72]. The activity decreases of more than 300-fold if the inhibitor is added during infection, thus suggesting that it inhibits viral entry. Moreover, nafamostat shows 30–240 nM concentration by iv administration through continuous infusion in DIC patients [72] and PK study in rats revealed the maximum concentration of intact nafamostat in the lung after infusion to be about 60-fold higher in comparison with the maximum blood concentration [73]; such an accumulation may partially suppress SARS-CoV-2 infection. A randomized clinical trial has recently been launched for adult COVID-19 patients to investigate the ability of nafamostat to slow down the lung disease (ClinicalTrials.gov Identifier: NCT04352400). Indeed, the efficacy of nafamostat as mucolytic and anticoagulant agent and the potent inhibitory activity against TMPRSS2 are useful features to improve the clinical conditions of hospitalized COVID-19 patients.

**Figure 12:**
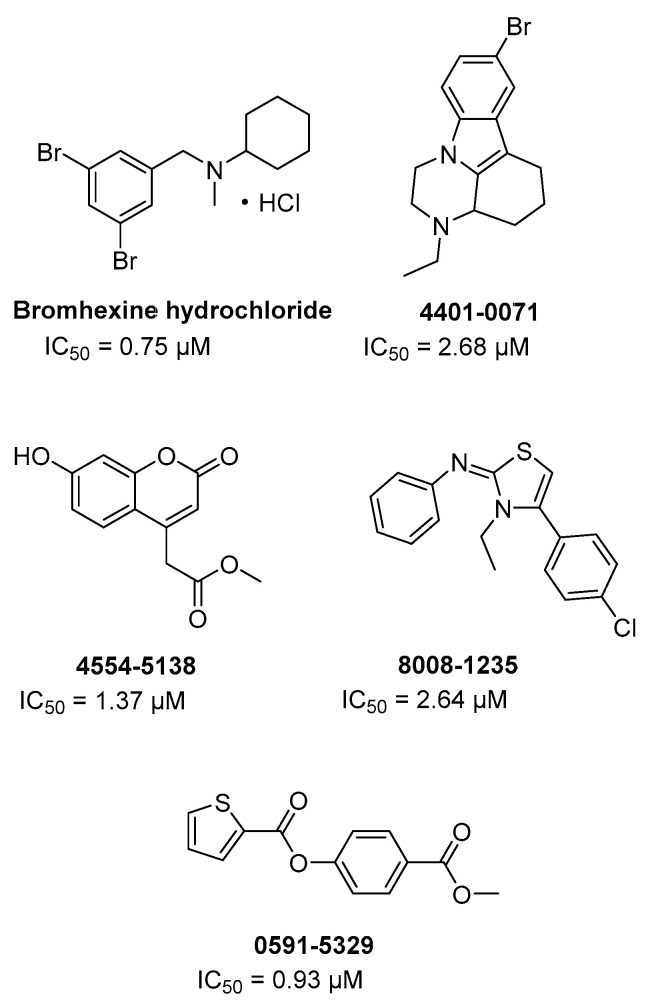
Chemical structures of Bromhexine hydrochloride and additional four hits identified by a biochemical HTS as TMPRSS2 inhibitors, with their inhibitory activity.

Through a HTS on FDA approved drugs and other commercial libraries (around 70 K cmpds) against TMPRSS2 in order to find new potential anti-metastatic agents for prostate cancer, bromhexine hydrochloride and other four hits (Figure 12) were identified as inhibitors of the enzyme at a concentration below 5 µM [74]. In particular, bromhexine hydrochloride exhibited the most potent inhibition with IC_50_ = 0.75 µM, resulting specific for TMPRSS2 being significantly less active (50–80-folds) against hepsin and matriptase and not active up to 100 µM against trypsin and thrombin. Moreover, the new repurposed drug was evaluated in cells and in rodent animals without showing significant toxicity. Bromhexine is structurally unrelated to guanidine derivatives camostat and nafamostat, and no data are provided on kinetics of inhibition and putative binding site. However, bromhexine is an orally bioavailable drug used as mucolytic cough suppressant and with no substantial adverse effects. Indeed, in China an interventional clinical trial for COVID-19 has recently been approved in order to evaluate the efficacy and safety of bromhexine hydrochloride in patients with suspected or novel coronavirus pneumonia (ClinicalTrials.gov Identifier: NCT04273763). The treatment is randomized open label, based on the administration of bromhexine hydrochloride tablets in combination with standard treatment for COVID-19 (Arbidol hydrochloride granules/Recombinant Human Interferon α2b spray). A larger clinical study on 140 participants, at early phase I, involves the treatment with bromhexine alone or in combination with hydroxychloroquine sulphate, in order to evaluate the effect of bromhexine in preventing the development of COVID-19 (ClinicalTrials.gov Identifier: NCT04340349). In summary, the above-described clinical trials aim to counteract the SARS-CoV-2 infectivity and, in this regard, bromhexine is investigated as a promising inhibitor of TMPRSS2.

**Figure 13:**
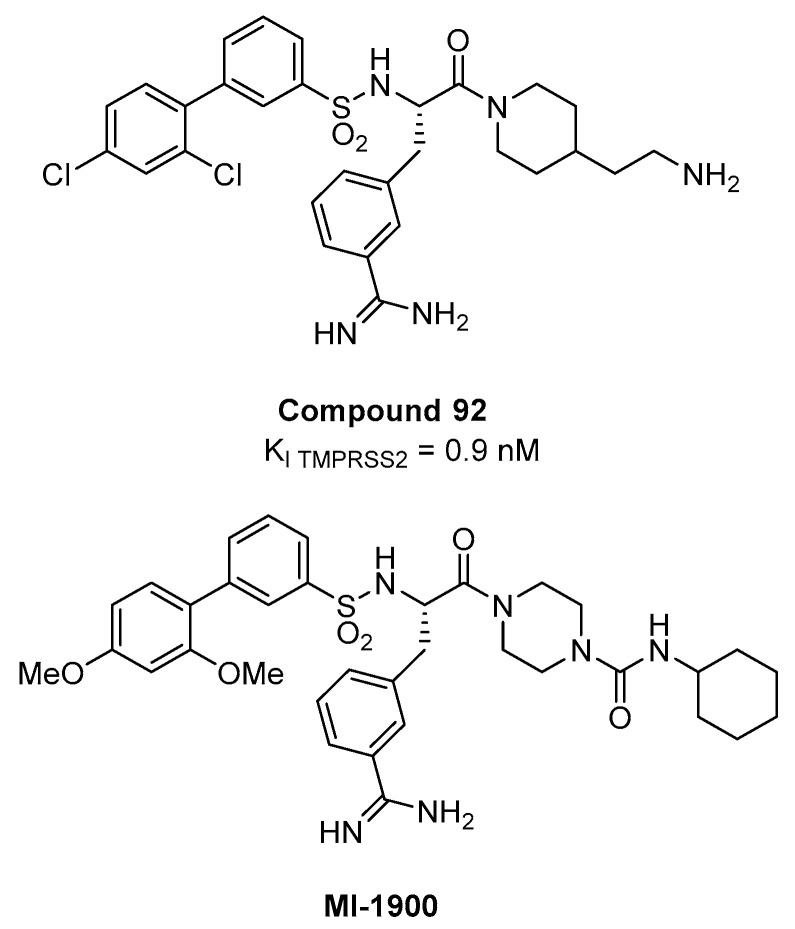
Substrate analogues containing 3-amidinophenylalanine as P1 residue as TMPRSS2 inhibitors compound 92 and MI-1900 showing antiviral activity against SARS-CoV-2.

A peptidomimetic approach represents a possible alternative towards the development of serine protease TMPRSS2 inhibitors. A series of 4-amidinobenzylamide derivatives, known as inhibitors of various trypsin-like serine protease, was screened against TMPRSS2 [75,76,77] in order to systematically investigate its substrate specificity [78], since the catalytic domain of all trypsin-like serine proteases share structural features and folding pattern. The screening revealed a preference for basic P3 residues in D-configuration, such as D-arginine, proline or glycine residues in P2 position, and a particular preference for 4-amidinophenylalanine amide as P1 residue. Upon SAR investigation, compound 92 (Figure 13), having the P1 *m*- amidinophenylalanine piperidine amide with a basic ethylamine chain extending towards the S1’ and a bulky biphenyl sulfonamidic *N*-cap, turned out to be the most potent inhibitor with a *K*_i_ value of 0.9 nM for TMPRSS2. In Calu-3 airway epithelial cells that were infected with human pandemic influenza viruses, 92 caused dose-dependent reduction in viral titers (10–100-fold at 10 µM and 100–1000-fold at 50 µM, after 24 h) without affecting cell viability. However, the significant discrepancies between the nM *K*_i_ on the isolated TMPRSS2 and activity in cell-based context observed at µM concentration are likely due to the high polarity of the compound that has two protonable groups at physiological pH. Recently, compound 92 and its less polar analogue MI-1900 have been shown to reduce by 25-fold virus titer in SARS-CoV-2 Calu-3 infected cells at a concentration of 10 µM, without showing toxicity up to 50 µM [79]. 

**Figure 14:**
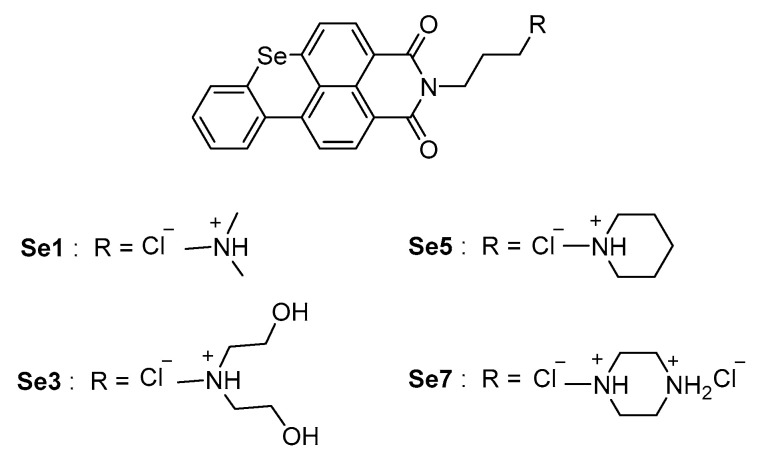
Chemical structures of Benzoselenoxanthene analogues, TMPRSS2 G-quadruplex stabilizers.

In recent times, it has been shown that a guanine-rich tract in the promoter region of human TMPRSS2 gene is able to form G-quadruplex secondary structures in the presence of potassium cations and adjust the gene transcription process [80]. Because the guanine-rich sequence has proven relevant for TMPRSS2 promoter activity [80], the use of compounds that are capable of stabilizing G-quadruplex structures and thus reducing/blocking transcription of the TMPRSS2 gene has been proposed as a potential host targeting strategy. Seven benzoselenoxanthene analogues were designed and synthesized for this purpose, and the compounds Se1, Se3, Se5, and Se7 (Figure 14) have shown to increase the stability of the TMPRSS2 G-quadruplex in vitro, corresponding to an effective decrease of TMPRSS2-gene expression in Calu-3 cells. At a later stage, Shen et al. evaluated the inhibition of viral propagation in Calu-3 cells infected with Influenza A virus. Benzoselenoxanthene analogues led to a near-complete reduction of virus titer at a concentration of 8 µM, with an antiviral activity comparable with anti-influenza drug Oseltamivir, although definitely inferior to camostat inhibitor. Therefore, no significant cytotoxic effects have been revealed at 10 µM. In summary, the down-regulation of TMPRSS2 expression through G-quadruplex structure stabilization is affirmed as a promising strategy for the inhibition of viral infection and represents a pioneering starting point for novel drugs against SARS-CoV-2.

### 3.2. CatB/L as Host Targets and Its Inhibitors

As described above, SARS-CoV-2 enters cells also exploiting the endosomal pathway, mediated by CatB/L and their inhibition for the treatment of SARS-CoV-2 infection may be a promising host antiviral strategy, as supported by previous literature on SARS-CoV-2 and related CoVs [21,62,70,81,82]. The CatB/L are human lysosomal cysteine proteases belonging to clan CA1 of the papain structure, which includes eleven subtypes of cathepsins (L (L1), B, C, F, H, K, O, V (L2), X, S, W). Whereas, the lysosomes are the cell organelles used for the recycling and degradation of biological molecules, as well as for the defense against external agents, the cathepsins participate in processes involving cell death, protein degradation, autophagy, and immune signaling, also depending on cell and tissue types [83]. The aberrant dysregulation of these functions is the basis of various pathologies, such as cancer and metastasis, rheumatoid arthritis, diabetes, immunological responses, and inflammatory respiratory disease, to cite a few, thus these proteins are important targets within several drug discovery fields [84,85,86,87]. The most challenging aspect in targeting cathepsins is the achievement of protein selectivity in order to avoid toxicity and side effect that could derive from broad inhibition of many different cathepsins. Previous studies have demonstrated that various viruses, such as SARS-CoV and other CoVs [62,88], Ebola virus, Hendra virus, and Nipah virus, require specifically CatL for surface glycoprotein priming to activate the membrane fusion process [86,89]. Structural and mechanistic information are available on CatB/L and many inhibitors have been reported for different therapeutic areas [90]. CatB/L are cysteine endopeptidase characterized by a catalytic triad made-up by Cys25-His159-Asn175 residues. It is noteworthy that CatB is the unique cathepsin able to act also as exopeptidase (carboxypeptidases), due to a conformational change dependent on “occluding loop” of 20 amino acid (Ile105-Thr125). The occluding loop blocks the portion of primed site of substrates in the active site, in order to leave space for two amino acid residues of C-terminal and favor the correct positioning of the substrate by two His residues (His110 and His111) that bind the terminal carboxylate [91]. Although the active sites of CatB/L did not present substantial differences in terms of volume and shape, they show different substrate specificity [92]. The substrate specificity of carboxypeptidase CatB allows for diverse amino acids at P1 position, such as Leu, Ser, or Gly, with a preference for basic amino acid as Arg; S2 is large enough to accommodate Val and Phe as residues in P2 position; the P3 position prefers Ile residue then Lys, and Phe [92]. The presence of a Gly residue in P3 is strongly required for CatB endopeptidase specificity, whereas it is well suited to sterically small S3’ subsite due to occluding loop. It has been shown that CatL [93] prefers a small aliphatic amino acid at P1 position, such as Gly, while requires an aromatic residue at P2 position for specificity of substrate, and bulky flexible substituents at P3, with a preference for the amino acid Leu. The differences in substrate specificity of CatB/L are useful for the design of selective and most potent inhibitors towards each cysteine protease. Additionally, for cathepsins inhibitors, the design of reversible covalent inhibitors represents a preferential path by introducing electrophilic warheads aside P1 position, which trap the nucleophilic Cys25 residue in the active site of the enzyme. To date, numerous cathepsin inhibitors have been reported in publications and patents and can be classified according to warheads and mechanism of action, as already reviewed [87,90]. 

**Figure 15:**
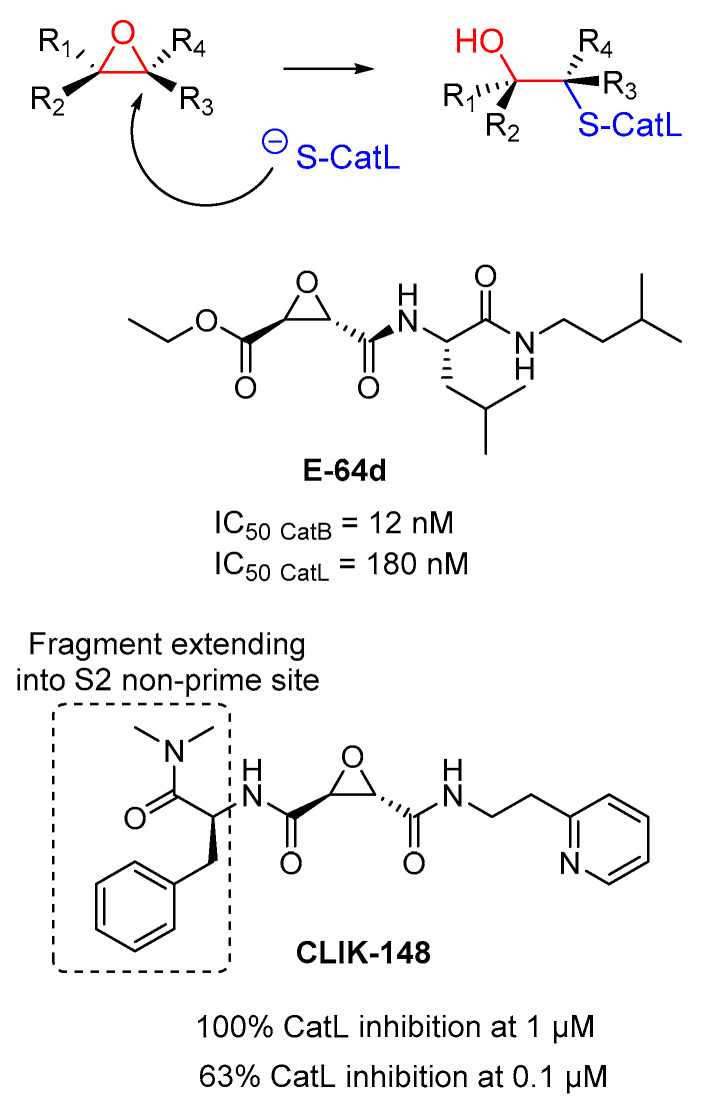
Structures and inhibitory activities of epoxysuccinates E-64d and CLIK-148 against human cathepins.

**Figure 16:**
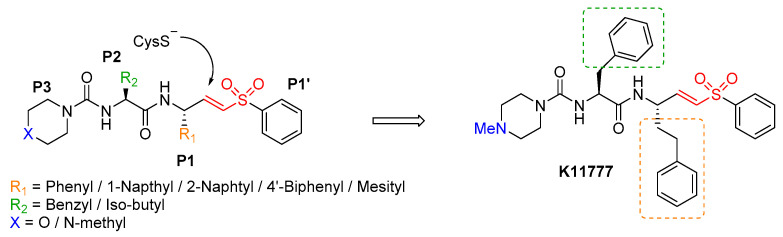
Peptidyl aryl vinylsulfone SAR (left) and the best compound K11777 as irreversible inhibitors of cathepsins and SARS-CoV replication in Vero 76 cells.

As anticipated above, the cathepsin inhibitor E64d has been found to enhance camostat SARS-CoV-2 entry inhibition and antiviral activity in cell lines, thus providing evidence to consider CatB/L as anti-CoV host targets [21]. However, single treatment with E64d only leads to partial inhibition, likewise Camostat. E-64d is an unselective and covalent cathepsin inhibitor, containing an epoxide ring as warhead that can undergo nucleophilic attack by Cys-SH and form the covalent bond at the catalytic site [94] (Figure 15). Therefore, the epoxysuccinyl-derivate is highly reactive and it has not the structural requirements to selectively target one protein, showing action against both cathepsin B and L and a moderate 24-fold selectivity over cathepsin H, with low toxic effects [95]. CoVs seem to preferentially engage CatL to entry into host cells [62,88], hence it may be more appropriate to selectively target CatL, although further studies should be performed to prove whether selective CatL inhibitors are more effective and safer than CatB/L inhibitors. However, the lack of selectivity of E-64d is attributable to the fact that it only occupies the prime-site at the enzyme pocket while of P2 site is determinant for selectivity towards the CatL [96]. For instance, the crystal structure of the papain/CLIK-148 complex (PDB ID: 1CVZ) [97] revealed that *N*-terminal pyridine-ring engages a π- π interaction with the Trp-177 in the prime site, while the rigidity and bulkiness of phenyl-ring fits well in the S2 non-prime site (Figure 16). CLIK-148 showed a preferential inhibition of CatL than the other cathepsins, achieved by fragments that extend into S2 non-prime site of the protease, thus representing a potential template for design of selective CatL inhibitors.

An attractive hit candidate for SARS, Ebola virus, and potentially for SARS-2 infection is the vinylsulfone-derivate K11777 (Figure 16), capable of inhibiting cathepsin-mediated viral entry. K11777 is known for inhibit cruzain, a CatL-like protease of the protozoan parasite Trypanosoma cruzi [98] and several human cathepsins [99] that has been under advanced stages of preclinical development for various parasitic diseases and has proven to be non-mutagenic with satisfactory safety and PK profiles in different animal models, such as rodents, dogs and primates [100,101,102,103]. K11777 is a covalent inhibitor of cysteine protease, bearing the vinylsulfone moiety, which acts as a Michael acceptor and reacts with Cys-SH in the catalytic pocket of the protease. In line with the proven efficacy against various cysteine proteases (Figure 16) [98,104,105,106]. SAR studies around 3-aryl vinyl sulfone analogues as inhibitors of CatB/L highlighted that a phenetyl group at the P1 is preferred for cathepsin L, while 2-napthyl group for cathepsin B, an aliphatic as in Leu or a benzyl group as in Phe are accepted by CatL, while CatB only tolerates aromatic residues [107]. In general, compounds with a 4-methylpiperazinyl group in position 3 were more active than compounds with a morpholinyl group. The basic piperazine ring at the P3 position was important likely to promoteen the accumulation into the lysosomal acidic environment, where the inhibitor could find its molecular target. K11777 having these chemical requirements potently inhibit CatB/L and has demonstrated activity against a variety of CoVs and filoviruses that engage host cathepsins for cellular entry via endosomal pathway [70]. Interestingly, K11777 inhibited SARS-CoV replication in cell based assays, with EC_50_ ranging from 0.05 to 0.52 µM, showing 90% reduction in viral yields with EC_90_ ranging from 0.35–1.04 µM, with low toxicity (CC_50_s > 50 µM, SIs ranging from 150.6 to > 2112), depending of the viral strain, the method to evaluate antiviral effect and the cell type (summarized in Table 1).

K11777 is an attractive candidate for host antiviral strategy and a potential starting point for the development of broad-spectrum therapy deserving evaluation against SARS-CoV-2. However, few medicinal chemistry efforts have been made around K11777 within the CoV research field, a small series of analogues synthesized in the same study only show modifications at the substituent on the nitrogen of the N-piperazine cap. No data against the isolated target are available, but data from cell-based assays indicate that compounds retaining basic piperazine moieties show potent activity comparable to K11777, while derivatives lacking of protonable group at physiological pH have a drop of 10–100-fold in activity. The basic piperazine ring at the P3 position is important for the efficient accumulation of the inhibitor in lysosomal compartment rather than for the interaction with cathepsins. Moreover, K11777 has a quite long story as preclinical candidate against protozoa, thus some DMPK information are available. It has been evaluated by oral administration in monkeys (50 mg/kg) and rodents (50 mg/kg) showing good metabolic stability (3–4 h), but moderate 15–20% of bioavailability, consistently to its peptidic nature, plasma levels at 1h in rodents ranging from 0–3 to 1 µM [103]. 

**Figure 17:**
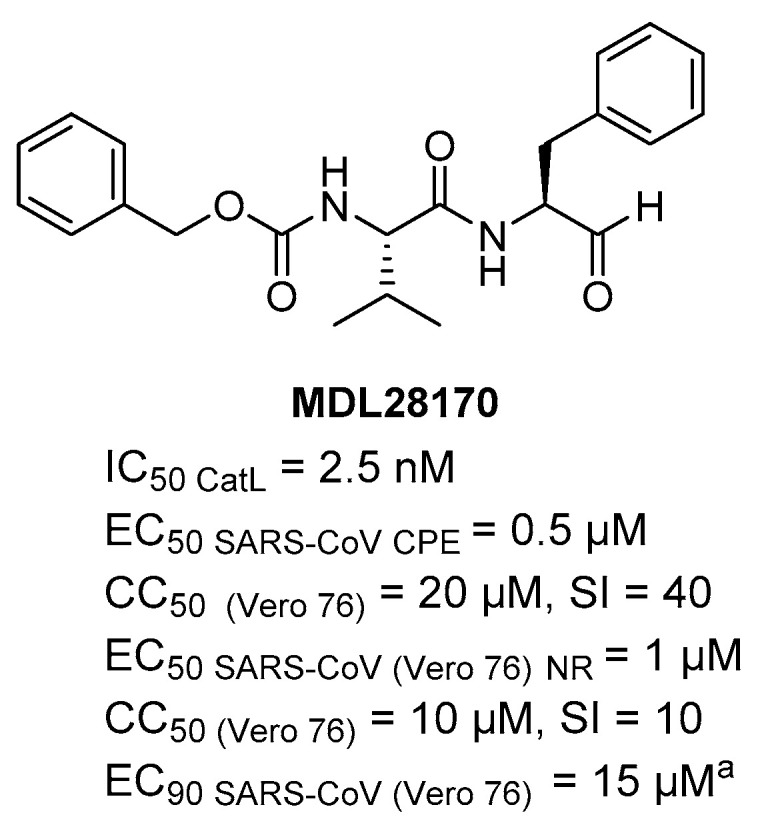
Chemical structure and biological activity of aldehyde MDL28170 as potent covalent reversible inhibitor of CatL and anti-CoV agents; ^a^ virus yield reduction assay (biological data from Ref. [108]).

In order to find compounds able to block SARS-CoV entry in host cells a biochemical screening against CatL was carried out using a library of 1000 cysteine proteases inhibitors and MDL28170 (Figure 17), a reversible covalent inhibitor with an aldehyde warhead (known also as calpain inhibitor III or Z-Val–Phe [CHO]), resulted able to exert potent inhibition of CatL with IC_50_ = 2.5 nM and to inhibit SARS-CoV replication, but in cell-based assay data (EC_50_ and CC_50_) are not available. Moreover, the inhibitor is able to block S protein-mediated entry of pseudo virions HIV-luc SARS S into host cells with IC_50_ < 100 nM [62]. In another study, Barnard DL et al. have reported the activity of MDL28170 in inhibiting cell culture infection of SARS-CoV showing less potent activity with EC_50_ ranging from 0.5 (CPE) to1 µM (neutral red) and slight toxicity with CC_50_ = 20 µM (CPE) and 10 µM (Neutral red). Unfortunately, in viral tritation the compound did not show selective antiviral effect [108]_._ MDL28170 iv dosed (50 mg/kg) in rodents preclinical model of ischemia showed a half-life of 2h, but additional DMPK data are not available [109,110]. However, it would also be interesting to evaluate its activity on SARS-CoV-2 to serve as starting hit for optimization.

**Figure 18:**
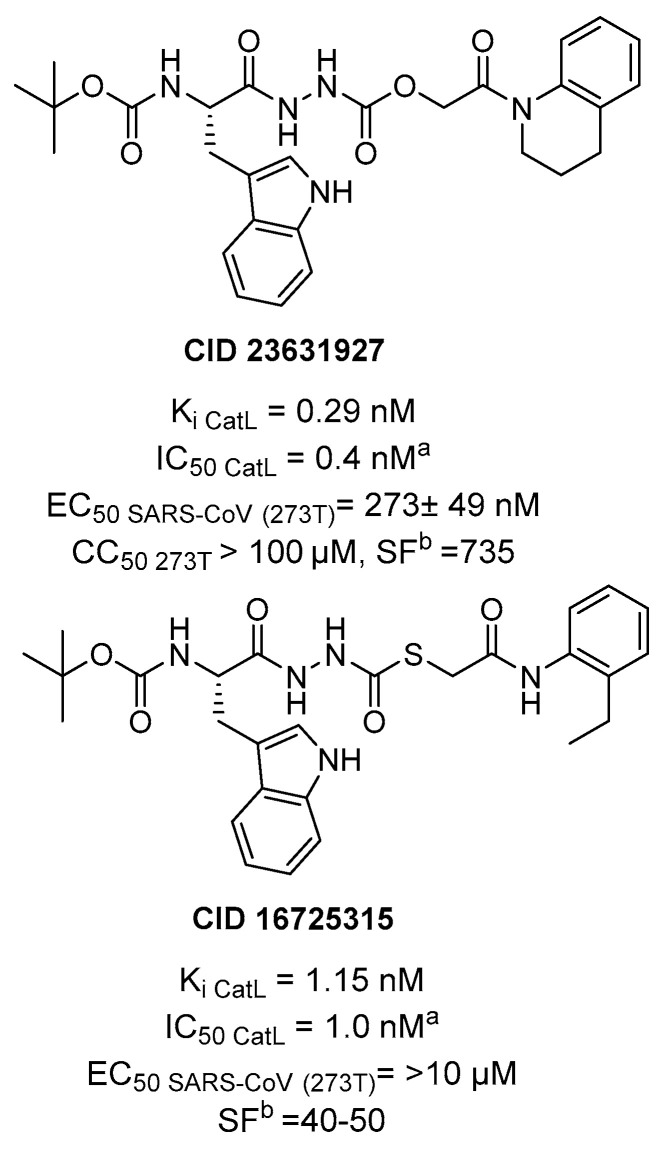
Structures and biological activities of tetrahydroquinoline oxocarbaze CID 23631927 and its analog CID 16725315 covalent reversible inhibitors of human CatL and anti-SARS-CoV-2 agents; ^a^ Preincubation for 4 h; ^b^ Cathepsin L/B selectivity ratio.

Although, to date, no CatL inhibitors are in clinical trials, unlike cathepsin B inhibitors, several compounds have shown selectivity of action towards CatL, sometimes with nM potency. For the purpose of this review, it is noteworthy to report a study focusing on a small-molecule oxocarbazate inhibitor CID23631927 that has shown a potent and selective antiviral activity against SARS-CoV in cell context, targeting CatL (Figure 18). Indeed, the tetrahydroquinoline oxocarbazate (PubChem) CID 23631927 was reported to potently inhibit CatL with a *K*_i_ = 0.29 nM, and IC_50_ = 0.4 nM (4 h pre-incubation), with a considerably high CatL/B selectivity ratio of 735. It is noteworthy that the oxocarbazate inhibitors are likely to act as covalent reversible inhibitors, because there is clear time-dependent inhibition and replacement of this moiety resulting in loss of activity; the mechanism of inhibition involves a tetrahedral intermediate by attack of the catalytic Cys25 on the oxocarbazate carbonyl [111]. The small-molecule oxocarbazate binding mode has been investigated by molecular modelling studies, suggesting that the tetrahydroquinoline fills the S1’ subsite, the *tert*-butoxycarbonyl group establishing hydrophobic interactions on the S3 subsite, while the indole group in *S* configuration binds to the S2 subsite, likely directing the specificity of the substrate [112]. Interestingly, derivative CID 23631927 was able to potently inhibit SARS-CoV entry in cellular (human embryonic kidney 293T) pseudotype model of infection (EC_50_ = 0.27 µM) and no toxicity up to 100 µM was observed in another cell type (human aortic endothelial cells). On the contrary, a close analogue (PubChem) CID 16726315 able to exert potent inhibition of CatL as well, did not have significant activity in cell lines, which was likely due to unsuitable chemico-physical properties across cell membrane. On the basis, of this promising background data, derivative CID 23631927 could be a promising candidate as tool compound to block SARS-CoV-2 infection as selective and potent inhibitor of human CatL in cell. 

## 4. Conclusions

The identification of antiviral agents against SARS-CoV-2 to treat COVID-19, but also against other CoVs to identify broad-spectrum inhibitors, is an urgent medical need. Researchers all around the World are exploiting different strategies targeting both viral and host factors making COVID-19 drug discovery an emerging, rapidly evolving, and intriguing research field. Besides targeting intracellular steps of viral replication, the inhibition of virus entry into host cells represents another way to prevent/treat viral infections. 

The inhibition of S/ACE2 interaction by targeting the S RBD region with peptides appears as a fast route to block viral entry, and a 23-mer peptide SBP1 with low nM affinity has been identified, but activity in cell-based assays is not known. However, the RBD region is the most variable among CoVs and is more prone to mutate, thus it is difficult to develop broad-spectrum inhibitors. In this scenario, fusion inhibitors targeting the more stable HRs are a more advantageous approach. In particular, HR2 sequence-based peptides could pave the way for the development of new therapeutics with broad-spectrum anti-CoVs activity. Additionally, the HR2 may likely have high resistance barrier to the viral mutations. Among the peptides reported, the lipopeptide EK1C4 shows potent S-mediated fusion inhibition coupled to selective broad antiviral activity in cell-based assays against SARS-CoV-2 and other relevant CoVs, such as MERS-CoV. Interestingly, the lipopeptide protect HCoV infected mice when administered from 0.5 to 4 h pre- and 0.5 h post-infection, holding promise to be developed as the first pan-CoV fusion inhibitor-based antiviral therapeutic to prevent/treat COVID-19 and other CoV diseases.

To inhibit viral entry, targeting host TMPRSS2 and CatB/L could be a promising approach to prevent/treat SARS-CoV-2 and parent CoVs infection. However, the identification of specific potent inhibitors of TMPRSS2 with suitable chemico-physical and PK properties is an emerging field and there is room for improvements. Of course, research efforts should also aim to better clarify substrate specificity and structural features of this enzyme thus allowing for more focused rational design of inhibitors. On the other hand, the availability of drugs already in use for other therapeutic areas that are able to exert potent and selective antiviral activity against SARS-CoV-2 and/or other CoVs likely targeting TMPRSS2, such as nafamostat, can be a candidate for drug repurposing and used as template for the design of new inhibitors. On the other hand, inhibition of cathepsins, especially CatL, can offer a promising host target approach to inhibit SARS-CoV-2 replication. The inhibitors of CatL already shown active against the related SARS-CoV also have the potential to inhibit SARS-CoV-2. The cruzain inhibitor K11777 and the preferential CatL inhibitor CID23631927 can represent suitable starting points to design selective and potent anti-CoV agents with optimized DMPK properties. Moreover, many CatL inhibitors have been reported in literature and they are characterized for the biological profiles; therefore, systematic analysis of the available information can be helpful within COVID-19 drug discovery.

## Figures and Tables

**Table 1:** Inhibitory activity of K11777 against SARS-CoV replication in Vero 76 cells.

Compound	SARS-CoV	CPE Inhibition			NR Assay			Virus Yield Reduction
		EC_50_ (µM)	CC_50_ (µM)	SI	EC_50_ (µM)	CC_50_ (µM)	SI	EC_90_ (µM)
K11777	Urbani	<0.05	>106.6	>2111	<0.52	>100	>193	0.35
	Toronto-2	<0.05	85.2	>1704	<0.35	52.7	>151	1.04

## Abbreviations

WHO	World Health Organization
CoV	Coronavirus
SARS-CoV-2	Severe acute respiratory syndrome 2
COVID-19	Coronavirus disease 2019
SARS-CoV	Severe acute respiratory syndrome virus
MERS-CoV	Middle East respiratory syndrome virus
ACE2	Angiotensin-converting enzyme 2
Cat	Cathepsin
TMPRSS2	Transmembrane Protease Serine Type 2 Secretory
S	Spike protein
RBD	Receptor Binding Domain
HB	Helix Bundle
E	Envelope
M	Membrane
N	Nucleocapsid
PDB	Protein Data Bank
